# Suicidality and gambling among young adults in Great Britain: results from a cross-sectional online survey

**DOI:** 10.1016/S2468-2667(20)30232-2

**Published:** 2021-01-05

**Authors:** Heather Wardle, Sally McManus

**Affiliations:** a School of Social and Political Sciences, University of Glasgow, Glasgow, UK; b Faculty of Public Health and Policy, London School of Hygiene & Tropical Medicine, London, UK; c School of Health Sciences, City, University of London, London, UK

## Abstract

**Background:**

Suicide rates in young people have increased in England and Wales since 2010. There are a range of possible explanations for this increase, and problem gambling has been suggested as a potential risk factor. We aimed to examine the association between suicidality (suicidal thoughts and suicide attempts) and problem gambling specifically for young adults in Great Britain, where gambling has become more widely available and normalised in the past two decades.

**Methods:**

We analysed data from the Emerging Adults Gambling Survey: a cross-sectional, online, non-probability sample survey of young adults aged 16–24 years living in Great Britain, who were selected from a YouGov online panel. Participants were eligible if they had not taken part in any other YouGov survey on gambling in the past year. We examined associations between problem gambling (defined as a score of 8 or higher on the Problem Gambling Severity Index [PGSI]) and suicidal thoughts and suicide attempts in the year before survey completion in a series of regression models, with and without adjustment for sociodemographic factors, alcohol use, video gaming, anxiety, loneliness, and impulsivity.

**Findings:**

3549 eligible participants completed the survey between June 25 and Aug 16, 2019. 24 (37·0% [95% CI 25·6–50·2]) of 62 men who had attempted suicide in the past year had survey scores that were indicative of problem gambling, compared with 38 (3·6% [2·6–5·0]) of 1077 men who had not attempted suicide or had suicidal thoughts in the past year. 13 (14·5% [8·5–23·6]) of 85 women who had attempted suicide in the past year had survey scores that were indicative of problem gambling, compared with 25 (2·0% [1·4–3·0]) of 1184 women who had not attempted suicide or had suicidal thoughts in the past year. The adjusted odds ratio for attempted suicide was 9·0 (4·1–19·7) in men with scores that indicated problem gambling and 4·9 (2·0–12·0) in women with scores that indicated problem gambling, compared with participants of the same gender with PGSI scores of 0.

**Interpretation:**

Problem gambling appears to be associated with suicide attempts in both young men and young women. This association persisted after adjusting for anxiety, impulsivity, life satisfaction, and other factors, which suggests that other mechanisms, such as the severity and multiplicity of harms experienced, or gambling to cope with life stressors, might underpin this relationship. Young people with problem-gambling behaviours should be considered at risk for suicidality.

**Funding:**

Wellcome Trust.

## Introduction

Suicide is one of the leading causes of death among young people worldwide.^1^ In Great Britain, as elsewhere, increased rates of suicide and self-harm in young people have led to increased concern among the public, policy makers, and clinicians.^2^, ^3^, ^4^, ^5^ Although many antecedents to suicide in young people are known,^6^ uncertainty remains about what factors are driving the temporal change in suicide rates. Some established risk factors are stable (eg, neglect and abuse) or decreasing in prevalence (eg, alcohol and drug misuse). Explanations postulated for the increase in suicide rates include the role of social media, online bullying, exam and lifestyle stress, and increasing insecurity among young people.^6^ However, other factors are probably also involved, and might be further compounded by the COVID-19 pandemic.^7^

Associations between problem gambling and suicidality among adults have been documented, showing that those with problem-gambling behaviours (both in treatment and community settings) are more likely to attempt suicide or have suicidal thoughts.^8^ However, few studies have examined this association specifically among young adults, and these were done before the advent of online gambling and the normalisation of gambling that has occurred in many jurisdictions.^9^, ^10^, ^11^, ^12^, ^13^ To our knowledge, two studies have examined the relationship between gambling and suicide among young adults using data collected since 2010. Both studies used non-probability samples and neither study disaggregated results by sex. One study had a sample of 143 people in the USA; the other was a study of 450 students in South Korea.^14^, ^15^

The evidence gap for young adults is surprising, given the extent to which young people's engagement in other health-harming behaviours has changed over the past 20 years. In England, for example, the prevalence of non-suicidal self-harm has tripled since the year 2000 among those aged 16–24 years, while the proportion of young adults drinking alcohol above weekly limits decreased from 43% in 2005 to 28% in 2015,^16^, ^17^ and since 2005 the proportion who smoke cigarettes^18^ or use recreational drugs^19^ has also decreased. Although overall gambling participation among 16–24 year-olds has reduced (largely due to the declining popularity of the UK National Lottery), participation in specific forms of gambling, like online betting, has increased.^20^ Online gambling is the largest growth sector in Great Britain and now accounts for more than one third of the total gambling market.^21^ Among young men, the increase in online gambling is notable, with rates of online betting increasing from 12% of men aged 16–24 years living in England and Scotland in 2012, to 17% in 2018.^20^, ^22^ Online betting and gambling have one of the highest levels of association with problem gambling, and in some jurisdictions are regulated more tightly because of their greater association with harm.^23^ Considering the shifts in risk-taking behaviours among young adults, there is an urgent need to examine recent data on gambling and suicidality.

**Research in context**

**Evidence before this study**

We searched titles and abstracts in PubMed on Jan 17, 2020, using the following search terms: (“suicid*” AND “gambling” OR “betting”) AND (“young people” OR “young adults” OR “emerging adults” OR “students” OR “adolescen*”), to identify articles published in English with no date restrictions. Available evidence on suicidality in young adults who gamble is scarce and mostly is from studies of high school, college, or university students, or self-selected samples. Previous analyses have been mainly bivariate in nature, showing an association between problem gambling and lifetime suicidality. Few studies have focused on suicidal behaviour in the past year or examined how this relationship might be affected by other factors. Even fewer studies have looked at the relationship separately for men and women, which is a notable gap in research given the gendered patterns of gambling and suicidality. Existing research is based on data which are now more than 10 years old, which were collected before the global increase in availability of gambling, and before the increase in suicidality in young people which has been reported in many countries.

**Added value of this study**

This study provides, to our knowledge, the first evidence of an association between problem gambling and suicide attempts in a large sample of young adults in Great Britain. Our findings show that the association is pronounced, evident in both genders, and remains after adjustment for other factors.

**Implications of all the available evidence**

The increased accessibility of gambling and increase in problem gambling might be a factor in the increased prevalence of suicidality in young adults in Great Britain and other countries. Early, easy, and persistent access to online gambling might have serious long-term public health implications. Young adults with problematic gambling behaviours should be recognised as being at risk for suicidality by clinicians, education professionals, and social services, as well as by the financial sector and those providing frontline welfare and debt advice. Although the availability of specialised treatment must expand to meet the existing need, national regulatory interventions and primary legislation should be considered by authorities to prevent harms.

Furthermore, studies have rarely examined the relationship between suicidality and gambling behaviour separately for young men and young women. Given the highly gender-specific nature of gambling engagement and experience of gambling problems, as well as different prevalence of suicidality among young men and young women, disaggregation by sex is important. One study which examined this relationship found that the association between suicidality and problem gambling only persisted for women when other factors like substance misuse, anxiety, and depression were taken into account.^9^ However, the study used data that were collected over 13 years ago and the prevalence and context of both gambling and suicidality in young people have now changed. Neither of the more recent studies had samples large enough for separate analyses of young men and young women.^14^, ^15^

We aimed to examine the association between current suicidality and problem gambling among men and women aged 16–24 years in Great Britain, and the effect of adjusting for factors such as engagement in social media, video gaming, alcohol use, anxiety, and common personality traits like impulsivity.

## Methods

### Data source and participants

We analysed data from the Emerging Adults Gambling Survey: a cross-sectional, online, non-probability sample survey for young adults. Participants were drawn from YouGov's online panel of more than one million people living in Great Britain.^24^, ^25^ This panel has up-to-date information on the profile of each member, allowing subsets of panel members to be invited to participate according to certain characteristics. For this study, participants were eligible if they were aged 16–24 years, lived in Great Britain, and had not taken part in any other YouGov study on gambling in the past year. Invitations to participate were sent by e-mail from YouGov to a random selection of their panel members, stratified by region. This invitation asked members to take part in a survey, without advertising its content, and asked participants to click through to the bespoke study. The first page of the bespoke survey described the aims and objectives of the survey and obtained informed consent. 3588 (93·0%) of 3856 people who accessed this page went on to complete the survey. Further waves of data collection from the same participants were planned for August and September, 2020 (data not yet available), and August, 2021, to examine individual trajectories over time; sample size calculations were done on the basis of being sufficient to estimate change in gambling prevalence between waves. Assuming a correlation between waves of 0·5, the study was able to detect changes in problem gambling behaviours of plus or minus 0·3 percentage points (at 80% power).

The survey included items on gambling, video gaming, social media, and health-related behaviours; it was developed by the lead author (HW) and reviewed by an expert panel. An online pilot survey collected data from 62 participants in May, 2019. Pilot survey responses were reviewed by the lead author and members of the YouGov team, and changes were agreed. The responses from the first 250 participants for the main data collection were further reviewed for consistency, routing accuracy, and to establish timing thresholds for seriousness checks. Participants who completed the survey in less than 1 SD of the mean completion time were excluded from our analyses; 39 participants were excluded for this reason.

The study protocol was registered,^26^ and ethical approval for the study was granted by the London School of Hygiene & Tropical Medicine's Ethics Review Panel (reference 16023).

### Procedures

Survey questions about suicidality were adapted from the Adult Psychiatric Morbidity Survey,^27^ and asked “In the last 12 months, have you ever thought of taking your life, even if you would not actually do it?” and “In the last 12 months, have you ever made an attempt to take your life, by taking an overdose of tablets or in some other way?”. Consistent with the approach adopted elsewhere,^28^ three variables were derived. One variable coded participants by whether they had experienced suicidal thoughts in the past year (yes or no); one coded whether they had made a suicide attempt in the past year (yes or no); and one combined variable with the following categories: those who had not thought about suicide or attempted suicide, those who had thought about suicide but not attempted it, and those who had attempted suicide.

Participants were asked to report whether they had ever gambled, and if so, how often they gambled, on a range of 17 different gambling activities that are legally available in Great Britain. Problem gambling was measured using the Problem Gambling Severity Index (PGSI): a validated tool for the identification of gambling problems.^29^ This was used for anyone who had gambled in the past year and produces estimates of current gambling problems. The PGSI score ranges from 0–27; a score of 0 indicates non-problem gambling or non-gambling; 1–2 indicates low-risk gambling; 3–7 indicates moderate-risk gambling; and a score of 8 or higher indicates problem gambling.

Impulsivity was measured using a shortened form of the Eysenck Impulsivity Inventory which is validated for use among adolescents aged 14–18 years.^30^, ^31^ Participants were asked to respond on a five-point scale to indicate how true seven different statements about impulsivity were for them. Impulsivity scores were computed as the average of the seven questions (mean 2·28 [SD 0·87]), which were similar to other published results among young people.^32^ Personal wellbeing was measured using the harmonised Office for National Statistics (ONS) four-item measure of personal wellbeing.^33^ This measure asks participants to rate on a scale of 0–10: their current levels of life satisfaction; whether they do things that they feel are worthwhile; how happy they felt yesterday, and how anxious they felt yesterday. Risky alcohol consumption (ie, of high risk to health) was identified using the modified Single Alcohol Screening Questionnaire.^34^ This questionnaire consists of one item from the Alcohol Use Disorders Identification Test about frequency of consuming 8 or more units of alcohol for men or 6 or more units of alcohol for women in a single event in the past year. A score of 3 or more identifies higher-risk drinkers. Perceived loneliness was assessed by one item from the Social Functioning Questionnaire.^35^ Participants were asked to assess with a four-category response (very much, sometimes, not often, or never) the extent to which they had felt “lonely and isolated from other people” in the past 2 weeks.

Participants were also asked how often they had played video games in the past year, coded into those who played video games at least once per week, or less than once per week week (including never). Participants were asked how much time they spent on social media on an average day, response options ranged from less than 0·5 h to 7 h or more per day, and were grouped into five categories ranging from less than 1 h to 5 h or more. Ethnicity was self-reported using the harmonised ONS ethnicity question. Because of low base sizes, responses were grouped into white or white British, Asian or Asian British, Black or Black British, and Mixed or Other. Age was captured by single year of age. Local-area level of deprivation was measured using English, Scottish, and Welsh indices of multiple deprivation scores matched at the output area and quintiled for analysis. Respondents were asked to report the level of academic attainment of both of their parents. This response was grouped by whether at least one parent had a degree or higher level of education, or whether both parent's qualifications were lower than degree level.

### Statistical analysis

All analyses were performed separately for each gender. Unadjusted associations between suicidality and gambling, video gaming, social media, demographics, socioeconomics, and personality and health-related factors were examined. Unadjusted odds ratios (ORs) were also calculated for suicidal thoughts, stratified by PGSI score.

Multivariable binary logistic regression analyses were run with suicide attempts as the dependent variable and PGSI score as the independent variable. Covariates were selected because of established or pre-hypothesised associations with both suicidality and gambling.^8^, ^36^, ^37^ To investigate whether other behaviours or traits explained the association between problem gambling and suicidality, groups of variables were added sequentially. Because suicide attempts are relatively rare events, models were constructed limiting the number of explanatory variables entered. Model one added ethnicity and local-area level of deprivation; model two added behavioural factors (ie, alcohol use, social media, and video gaming), model three added impulsivity, model four added perceived loneliness, and the fully adjusted model added personal wellbeing. Because the four personal wellbeing items were highly correlated, only life satisfaction and anxiety were included in the final model. However, entering all four items in different combinations did not substantially alter the results. With the exception of age, impulsivity scores, and personal wellbeing, all variables included in the models were categorical data. Missing data were minimal and participants with missing data were therefore excluded, except for data on ethnicity (missing for 159 participants), area deprivation (462 participants), and parental qualifications (207 participants), which were coded as dummy categories.

Diagnostic checks on multicollinearity were done by calculating the variance inflation factors of all independent variables; all variables had variance inflation factor values of less than 2, indicating that they were not too closely correlated.^38^ No adjustment was made for multiplicity. All bivariate analyses were done using the complex survey function in SPSS (version 20.0). These complex survey modules use an adjusted Wald *F* test as the default test of significance.^39^ This test assesses the extent to which the independent variable (eg, suicide attempts) varies by the dependent variables (eg, age or PGSI score), and is the test on which all p values are based. All multivariate analyses were done using the complex survey function in Stata 15 to adjust for weighted stratified survey design. All estimates were weighted to match the age, gender, and regional profile of 16–24 year-olds living in Great Britain. True (unweighted) base estimates and sample sizes are presented.

### Role of the funding source

The funder of the study had no role in the study design, data collection, data analysis, data interpretation, or writing of the report. The corresponding author had full access to all the data in the study and had final responsibility for the decision to submit for publication.

## Results

Data were collected from 3549 participants between June 25 and Aug 16, 2019. 85 (4·3% [95% CI 3·4–5·2]) of 1922 women and 62 (3·8% [3·0–4·9]) of 1627 men reported attempting suicide in the past year. 735 women (38·4% [36·1–40·8]) and 546 men (34·1% [31·7–36·5]) reported thinking about suicide in the past year. 730 men (45·5% [43·0–48·1]) had gambled in the past year, as had 766 women (39·4% [37·1–41·7]). The most common forms of gambling were purchasing scratch-cards (365 women [19·3%, 95% CI 17·5–21·3] and 297 men [18·8%, 16·9–20·9]), followed by playing lotteries (354 women [17·7%, 16·0–19·5] and 275 men [17·4%, 15·6–19·4]). Among men, online betting on sports events was the next most prevalent form of gambling (260 men [16·2%, 14·5–18·3]), whereas among women, playing bingo was the next most common form (142 women [7·0%, 5·9–8·3]), although 122 women (6·0%, 5·0–7·2) also engaged in online betting. In terms of PGSI scores, 80 men (4·9% [95% CI 3·9–6·1]) and 51 women (2·6% [1·9–3·4]) had scores that indicated problem gambling (table 1).

**Table 1:** Suicidality and gambling status, by gender

	**Neither suicidal thoughts nor attempted suicide**	**Suicidal thoughts only**	**Attempted suicide**	**Total**
**Men**				
Non-problem gambling (PGSI score 0)	861 (79·9% [77·3–82·3])	387 (79·1% [75·0–82·6])	33 (54·6% [41·6–67·0])	1281 (78·7% [76·5–80·7])
Low-risk gambling (PGSI score 1–2)	124 (11·8% [9·9–14·0])	56 (12·1% [9·3–15·6])	3* (4·8% [1·6–13·6])	183 (11·6% [10·1–13·4])
Moderate-risk gambling (PGSI score 3–7)	54 (4·7% [3·5–6·2])	27 (5·3% [3·6–7·7])	2* (3·7% [1·0–13·0])	83 (4·8% [3·9–6·0])
Problem gambling (PGSI score ≥8)	38 (3·6% [2·6–5·0])	18 (3·5% [2·2-5·6])	24 (37·0% [25·6–50·2])	80 (4·9% [3·9–6·1])
**Women**				
Non-problem gambling (PGSI score 0)	1041 (87·3% [85·1–89·3])	557 (85·8% [82·8–88·4])	64 (76·7% [66·4–84·5])	1662 (86·4% [84·6–87·9])
Low-risk gambling (PGSI score 1–2)	91 (8·2% [6·6–10·1])	74 (10·9% [8·6–13·6])	4* (4·4% [1·7–11·1])	169 (8·9% [7·6–10·4])
Moderate-risk gambling (PGSI score 3–7)	27 (2·5% [1·7–3·7])	9 (1·3% [0·7–2·4])	4* (4·5% [1·7–11·2])	40 (2·2% [1·6–3·0])
Problem gambling (PGSI score ≥8)	25 (2·0% [1·4–3·0])	13 (2·1% [1·2–3·6])	13 (14·5% [8·5–23·6])	51 (2·6% [1·9–3·4])

Data are n (column % [95% CI]). All percentage estimates have survey weights applied, while n represent the absolute unweighted number. Suicidality refers to suicidal thoughts and suicide attempts in the year before survey completion.

* Recommend using caution due to small cell sizes. PGSI=Problem Gambling Severity Index.

Problem gambling (PGSI score of 8 or higher) was more common in men who had attempted suicide in the past year than in men who had not attempted suicide or had suicidal thoughts (24 [37·0%, 95% CI 25·6–50·2] of 62 men *vs* 38 [3·6%, 2·6–5·0] of 1077 men). Similarly, problem gambling was more common in women who had attempted suicide in the past year than in women who had not attempted suicide or had suicidal thoughts (13 [14·5%, 8·5–23·6] of 85 women *vs* 25 [2·0%, 1·4–3·0] of 1184 women). The proportion of participants with scores indicating problem gambling were similar among those who had thought about suicide but had not made any suicide attempts in the past year, to those who had neither had suicidal thoughts nor made a suicide attempt in the past year (table 1).

Unadjusted regressions indicated that, for men, problem gambling was not associated with suicidal thoughts in the past year, and this relationship was at the margins of significance for women (p=0·041). However, problem gambling was strongly associated with suicide attempts for both men and women. The unadjusted OR for attempted suicide for those who had PGSI scores of 8 or higher indicating problem gambling compared with those who had PGSI scores of 0–7 indicating non-problem gambling was 15·1 (95% CI 8·2–27·9) in men and 8·0 (4·0–16·0) in women (table 2). For both women and men, impulsivity, perceived loneliness, low life satisfaction, and anxiety were all associated with suicide attempts. Mixed race or Other ethnicity and video gaming were also associated with suicide attempts in women (table 3).

**Table 2:** Unadjusted odds ratios (ORs) for suicidality by gambling status

	**Suicidal thoughts only**		**Attempted suicide**	
	Men	Women	Men	Women
Non-problem gambling (PGSI score 0)	1 (ref)	1 (ref)	1 (ref)	1 (ref)
Low-risk gambling (PGSI score 1–2)	1·1 (0·7–1·5)	1·4 (1·0–2·0)	0·6 (0·2–2·0)	0·5 (0·2–1·5)
Moderate-risk gambling (PGSI score 3–7)	1·1 (0·7–1·9)	0·5 (0·2–1·0)	1·1 (0·2–4·8)	2·5 (0·8–7·3)
Problem gambling (PGSI score ≥8)	0·6 (0·4–1·1)	0·7 (0·4–1·4)	15·1 (8·2–27·9)	8·0 (4·0–16·0)
p value	0·41	0·041	<0·0001	<0·0001

Data are OR (95% CI), or p values. Suicidality refers to suicidal thoughts and suicide attempts in the year before survey completion. PGSI=Problem Gambling Severity Index.

**Table 3:** Unadjusted odds ratios (ORs) for attempted suicide in the past year

	**Men**		**Women**	
	n (%)	OR (95% CI)	n (%)	OR (95% CI)
**Age**				
Mean, years (SD)	19·9 (2·3)	1·0 (0·9–1·1)	20·6 (2·4)	1·0 (0·9–1·1)
p value	..	0·77	..	0·54
**Ethnicity**				
White	1307 (80·3%)	1 (ref)	1605 (83·5%)	1 (ref)
Mixed or Other	81 (5·0%)	3·0 (1·3–6·9)	81 (4·2%)	3·9 (1·9–8·3)
Asian or Black	165 (10·1%)	1·4 (0·6–3·3)	151 (7·9%)	0·7 (0·2–2·1)
Missing	74 (4·6%)	2·1 (0·8–5·6)	85 (4·4%)	0·7 (0·2–2·4)
p value	..	0·071	..	0·0028
**Employment status**				
Employed, in education, or in training	1401 (86·1%)	1 (ref)	1706 (88·9%)	1 (ref)
Not in employment, education, or training	226 (13·9%)	1·0 (0·5–2·2)	215 (11·1%)	1·3 (0·7–2·5)
p value	..	0·95	..	0·41
**Local-area level of deprivation**				
Not in most deprived quintile	1039 (63·9%)	1 (ref)	1431 (74·5%)	1 (ref)
Most deprived quintile	245 (15·1%)	2·7 (1·4–4·9)	379 (19·4%)	1·3 (0·8–2·2)
Missing	343 (21·1%)	12·8 (0·6–2·6)	119 (6·2%)	0·3 (0·1–1·6)
p value	..	<0·0068	..	0·19
**Parents' qualifications**				
Degree-level or higher	935 (57·5%)	1 (ref)	109 (56·9%)	1 (ref)
Lower than degree-level or none	588 (56·1%)	1·1 (0·6–1·8)	727 (37·8%)	1·8 (1·1–2·9)
Missing	104 (6·4%)	0·5 (0·1–2·2)	103 (5·3%)	1·4 (0·5–3·7)
p value	..	0·61	..	0·056
**Alcohol use**				
Non-drinker	489 (30·1%)	1 (ref)	466 (24·2%)	1 (ref)
M-SASQ score 0–2	908 (55·8%)	0·9 (0·5–1·7)	1201 (62·4%)	0·9 (0·5–1·7)
M-SASQ score ≥3	230 (14·1%)	1·7 (0·8–3·7)	255 (13·2%)	1·7 (0·9–3·4)
p value	..	0·16	..	<0·12
**Impulsivity**				
Mean impulsivity score (SD)	2·3 (0·9)	2·4 (1·9–3·2)	2·2 (0·9)	2·5 (1·9–3·2)
p value	..	0·0001	..	<0·0001
**Social media use**				
<1 h per day	261 (16·0%)	1 (ref)	220 (11·5%)	1 (ref)
1 to <2 h per day	352 (21·6%)	0·5 (0·2–1·2)	326 (17·0%)	0·8 (0·3–2·1)
2 to <3 h per day	324 (19·9%)	0·4 (0·2–1·0)	402 (20·9%)	0·7 (0·3–1·7)
3 to < 5 h per day	283 (17·4%)	0·9 (0·4–2·0)	429 (22·3%)	1·6 (0·7–3·7)
≥5 h per day	407 (25·0%)	0·7 (0·4–1·6)	545 (28·4%)	1·0 (0·4–2·3)
p value	..	0·32	..	0·14
**Frequency of video-game play**				
Less than weekly or none	552 (33·9%)	1 (ref)	1265 (65·8%)	1 (ref)
Weekly or more	1075 (66·1%)	0·9 (0·5–1·6)	657 (34·1%)	2·1 (1·3–3·3)
p value	..	0·77	..	0·0006
**Perceived loneliness**				
Not at all	302 (18·6%)	1 (ref)	263 (13·7%)	1 (ref)
Not often	500 (30·7%)	4·5 (0·6–36·4)	515 (26·8%)	1·1 (0·3–4·9)
Sometimes	628 (38·6%)	13·6 (1·8–100·5)	889 (46·3%)	2·5 (0·6–9·6)
Very much	197 (11·2%)	34·0 (4·5–255·3)	255 (13·3%)	12·8 (3·3–49·5)
p value	..	<0·0001	..	<0·0001
**Life satisfaction**				
Mean life satisfaction score (SD)	7·7 (2·1)	0·8 (0·7–0·9)	7·7 (1·9)	0·8 (0·7–0·9)
p value	..	0·0070	..	<0·0001
**Anxiety**				
Mean anxiety score (SD)	5·3 (2·8)	1·4 (1·3–1·5)	6·0 (2·7)	1·3 (1·1–1·4)
p value	..	<0·0001	..	<0·0001

M-SASQ=modified single alcohol screening questionnaire.

For both men and women, problem gambling remained strongly associated with suicide attempts in the fully adjusted models. The adjusted OR for attempted suicide for those who had PGSI scores of 8 or higher indicating problem gambling compared with those who had scores indicating non-problem gambling (PGSI score 0–7) was 9·0 (95% CI 4·1–19·7) in men and 4·9 (2·0–12·0) in women (table 4). The final adjusted models also showed that impulsivity and perceived loneliness were associated with suicide attempts in the past year for both men and women, with higher adjusted ORs among those reporting “very much” loneliness or higher impulsivity scores. Among men, the adjusted OR for suicide attempts increased as self-reported anxiety increased. Among women, the adjusted OR for attempted suicide was higher in those who were Mixed race or Other ethnicity (table 4).

**Table 4:** Adjusted odds ratios (ORs) for attempted suicide in the past year

		**Model one**	**Model two**	**Model three**	**Model four**	**Model five**
**Men**						
Gambling status						
	Non-problem gambling (PGSI score 0)	1 (ref)	1 (ref)	1 (ref)	1 (ref)	1 (ref)
	Low-risk gambling (PGSI score 1–2)	0·6 (0·2–2·0)	0·5 (0·2–1·9)	0·5 (0·2–1·8)	0·5 (0·1–1·9)	0·5 (0·2–1·8)
	Moderate-risk gambling (PGSI score 3–7)	1·1 (0·3–5·0)	1·0 (0·2–4·9)	0·9 (0·2–4·3)	0·9 (0·2–4·1)	0·8 (0·2–3·9)
	Problem gambling (PGSI score ≥8)	12·9 (6·8–24·4)	12·9 (6·8–24·7)	7·9 (4·0–14·8)	8·9 (4·5–17·7)	9·0 (4·1–19·7)
	p value	<0·0001	<0·0001	<0·0001	<0·0001	<0·0001
Ethnicity						
	White	1 (ref)	1 (ref)	1 (ref)	1 (ref)	1 (ref)
	Mixed or Other	1·9 (0·7–5·0)	1·9 (0·7–5·2)	1·9 (0·7–5·0)	2·1 (0·7–6·2)	2·6 (0·9–7·2)
	Asian or Black	1·0 (0·4–2·3)	1·1 (0·5–2·7)	1·1 (0·5–2·7)	1·1 (0·5–2·7)	1·1 (0·4–2·6)
	Missing	1·5 (0·5–4·3)	1·6 (0·6–4·4)	1·5 (0·5–4·3)	1·4 (0·4–4·5)	1·4 (0·4–4·7)
	p value	0·58	0·54	0·57	0·53	0·35
Local-area level of deprivation						
	Not in most deprived quintile	1 (ref)	1 (ref)	1 (ref)	1 (ref)	1 (ref)
	Most deprived quintile	2·0 (1·1–3·9)	2·1 (1·1–4·0)	2·1 (1·1–4·0)	2·5 (1·2–4·9)	2·2 (1·1–4·3)
	Missing	1·0 (0·5–2·1)	1·1 (0·5–2·4)	1·1 (0·5–2·5)	1·2 (0·5–2·6)	1·2 (0·6–2·6)
	p value	0·081	0·073	0·077	0·034	0·092
Alcohol use						
	Non-drinker	..	1 (ref)	1 (ref)	1 (ref)	1 (ref)
	M-SASQ score 0–2	..	1·3 (0·6–2·7)	1·4 (0·7–3·0)	1·2 (0·6–2·5)	1·3 (0·6–2·8)
	M-SASQ score ≥3	..	2·0 (0·9–4·8)	1·9 (0·8–4·5)	1·9 (0·8–4·4)	2·1 (0·9–4·9)
	p value	..	0·23	0·31	0·12	0·23
Frequency of video-game play						
	Weekly or more	..	1 (ref)	1 (ref)	1 (ref)	1 (ref)
	Less than weekly or none	..	0·8 (0·5–1·4)	0·7 (0·4–1·3)	0·6 (0·4–1·1)	0·7 (0·4–1·2)
	p value	..	0·42	0·29	0·11	0·15
Impulsivity						
	Impulsivity score	..	..	1·9 (1·4–2·6)	1·7 (1·2–2·3)	1·5 (1·1–2·1)
	p value	..	..	<0·0001	<0·0001	0·0018
Perceived loneliness						
	Never	..	..	..	1 (ref)	1 (ref)
	Not often	..	..	..	3·4 (0·4–27·9)	3·0 (0·4–24·7)
	Sometimes	..	..	..	8·9 (1·2–65·2)	6·5 (0·9–47·8)
	Very much	..	..	..	28·4 (3·9–210·1)	15·3 (2·0–119·6)
	p value	..	..	..	<0·0001	0·0011
Life satisfaction						
	Life satisfaction score	..	..	..	..	0·9 (0·8–1·1)
	p value	..	..	..	..	0·29
Anxiety						
	Anxiety score	..	..	..	..	1·2 (1·1–1·4)
	p value	..	..	..	..	0·0003
**Women**						
Gambling status						
	Non-problem gambling (PGSI score 0)	1 (ref)	1 (ref)	1 (ref)	1 (ref)	1 (ref)
	Low-risk gambling (PGSI score 1–2)	0·6 (0·2–1·7)	0·5 (0·2–1·5)	0·5 (0·2–1·5)	0·4 (0·1–1·4)	0·5 (0·1–1·4)
	Moderate-risk gambling (PGSI score 3–7)	2·6 (0·9–7·7)	2·4 (0·8–7·2)	1·6 (0·5–5·1)	1·8 (0·6–4·8)	1·8 (0·7–4·7)
	Problem gambling (PGSI score ≥8)	7·8 (3·7–16·3)	7·2 (3·4–15·2)	3·5 (1·6–7·6)	4·9 (2·0–11·9)	4·9 (2·0–12·0)
	p value	<0·0001	<0·0001	0·0051	0·0013	0·0020
Ethnicity						
	White	1 (ref)	1 (ref)	1 (ref)	1 (ref)	1 (ref)
	Mixed or Other	3·7 (1·7–8·5)	4·1 (1·8–9·2)	4·4 (19–10·3)	5·6 (2·4–13·0)	6·0 (2·5–14·2)
	Asian or Black	0·7 (0·2–2·0)	0·8 (0·3–2·3)	0·6 (0·2–2·0)	0·5 (0·1–1·9)	0·5 (0·2–2·0)
	Missing	0·7 (0·2–2·1)	0·8 (0·3–2·2)	0·8 (0·3–2·5)	1·1 (0·3–3·6)	1·0 (0·3–3·5)
	p value	0·0097	0·0064	0·0053	0·0007	0·0006
Local-area level of deprivation						
	Not in most deprived quintile	1 (ref)	1 (ref)	1 (ref)	1 (ref)	1 (ref)
	Most deprived quintile	1·3 (0·8–2·2)	1·2 (0·7–2·0)	1·1 (0·6–1·8)	1·1 (0·6–1·9)	1·2 (0·7–2·0)
	Missing	0·3 (0·1–1·5)	0·3 (0·1–1·4)	0·3 (0·1–1·4)	0·4 (0·1–1·8)	0·4 (0·1–1·9)
	p value	0·17	0·23	0·31	0·41	0·41
Alcohol use						
	Non-drinker	..	1 (ref)	1 (ref)	1 (ref)	1 (ref)
	M-SASQ score 0–2	..	1·2 (0·7–2·1)	1·0 (0·6–1·9)	0·9 (0·5–1·6)	0·9 (0·5–1·6)
	M-SASQ score ≥3	..	2·0 (1·0–3·8)	1·2 (0·6–2·4)	1·0 (0·5–2·6)	1·0 (0·5–2·2)
	p value	..	0·12	0·86	0·87	0·90
Frequency of video-game play						
	Weekly or more	..	1 (ref)	1 (ref)	1 (ref)	1 (ref)
	Less than weekly or none	..	2·0 (1·3–3·3)	1·9 (1·2–3·1)	1·6 (1·0–2·7)	1·6 (0·9–2·7)
	p value	..	0·0038	0·010	0·065	0·078
Impulsivity						
	Impulsivity score	..	..	2·3 (1·7–3·0)	2·0 (1·5–2·6)	1·9 (1·4–2·6)
	p value	..	..	<0·0001	<0·0001	<0·0001
Perceived loneliness						
	Never	..	..	..	1 (ref)	1 (ref)
	Not often	..	..	..	1·4 (0·3–5·6)	1·4 (0·3–5·9)
	Sometimes	..	..	..	2·3 (0·6–8·6)	2·0 (0·5–7·6)
	Very much	..	..	..	12·4 (3·3–46·6)	8·3 (2·0–35·1)
	p value	..	..	..	<0·0001	<0·0001
Life satisfaction						
	Life satisfaction score	..	..	..	..	0·9 (0·8–1·0)
	p value Life satisfaction score	..	..	..	..	0·13
Anxiety						
	Anxiety score	..	..	..	..	1·1 (0·9–1·2)
	p value	..	..	..	..	0·29

PGSI=Problem Gambling Severity Index. M-SASQ=modified single alcohol screening questionnaire.

## Discussion

To our knowledge, this is the largest study of gambling behaviours and suicidality among young adults aged 16–24 years that has been reported in the past decade. Our results show the substantial association between problem gambling and suicide attempts, with the prevalence of suicide attempts increasing sharply with problem gambling. This association was identified in both young men and young women and represents the most recent evidence on the relationship between gambling and suicide since the advent and normalisation of online gambling.

Gambling, substance misuse, and suicide attempts are all forms of risky behaviour. All have undergone temporal shifts in prevalence among young people. In young adults in England, alcohol use has fallen, suicide attempts have increased, and the nature of gambling has shifted both in terms of location (increasingly online) and accessibility (available 24 h a day).^2^, ^16^, ^17^ It has been postulated that broader online behaviours might underlie increased suicidality among young people. Given that gambling is increasingly an online activity, one might assume that broader engagement in online activities could attenuate or explain the association between problem gambling and suicidality. However, our results suggest that this assumption is not correct, as adjusting for online video gaming did little to attenuate the association between gambling and suicide attempts.

For other risky behaviours, it has been suggested that a common aetiology underlies these associations with suicidality, such as certain personality traits like impulsivity. Using longitudinal data, Mars and colleagues^40^ found that those with problem alcohol or drug use were more likely to progress from suicidal thoughts to suicide attempts. Although our study is cross-sectional, we found a strong association between problem gambling and suicide attempts only. However, it remains to be seen if those experiencing suicidal thoughts who are problem gamblers are more likely to progress to suicide attempts. Future waves of this study could assess this.

The factor with the greatest attenuation of the association was impulsivity scores; adding impulsivity into the regression models led to substantial reductions in the ORs for attempting suicide. Impulsivity could explain in part the association between problem gambling and suicide attempts for some young people, but the association is not explained by impulsivity alone. In the fully adjusted model, the ORs for attempting suicide were still approximately nine times higher for men with problem-gambling behaviours and five times higher for women with problem-gambling behaviours compared with those with a PGSI score of 0. These ORs are substantial.

This study adds to an emerging breadth of evidence suggesting that problem gambling might be a major and under-recognised risk factor for suicidality. Similar results are evident for adults,^8^ where the relationship between problem gambling and suicidality was attenuated but remained when common mental disorders, substance misuse, and other health behaviours were taken into account. These previous results provided evidence that problem gambling should be viewed as a risk factor for suicidality, and that it is plausible that the harms experienced from problematic gambling might lead some people to consider taking their own lives.

YouGov is a non-probability panel, with serious attendant issues of generalisability. However, when researching young people, it has advantages in terms of sample coverage over probability methods; surveys using the postcode address file as a sample frame exclude many young people living in student halls of residence and surveys of students using sampling frames from university registers exclude those not in education, so results might not be generalisable to non-student populations. The YouGov panel includes both students and non-students, and the Emerging Adults Gambling Survey provides a closer estimate to national statistics of the proportion of young people not in education, employment, or training than so-called gold-standard probability surveys such as the Health Survey for England (appendix p 1). Both survey approaches appear equally likely to under-represent young men. The YouGov panel fails to capture fully those without internet access and web-enabled devices, although the effect of this is less on a survey of young people, because the digital exclusion is less pronounced than it would be for older age groups. An online survey might also overrepresent people who spend more time online, including those who engage more with online gambling.

Limitations of this study include that participants were recruited from a high-quality, but non-probability online panel, with issues of generalisability. Non-probability panel surveys tend to produce higher estimates of risk-taking behaviours than random-probability surveys.^41^ For young people in the UK, no sample approach has perfect coverage; it is unclear whether household surveys underestimate risk-taking behaviours or non-probability panel surveys overestimate them. However, the problem gambling prevalence figures collected in the Emerging Adults Gambling Survey are substantially higher than the national estimates produced by household studies. The rates of suicide attempts recorded are also higher than the most recent available national estimates (from 2014), although they are broadly in keeping with expectations of what trends might be found when these national estimates are updated in 2022 (appendix p 2). For these reasons, we recommend using caution when reviewing univariate results, and these data should not be viewed as representing the prevalence of suicidal behaviours among this age group. However, analysis has shown that non-probability closed online panels tend to produce similar conclusions to probability methods when focusing on multivariate analyses and when exploring the relationship between variables,^41^ which was the primary aim of this study. This analysis of non-probability panels, along with our findings being commensurate with data from probability studies among all adults,^8^, ^37^ can give increased confidence in our findings.

The Emerging Adults Gambling Study is an in-depth study of gambling behaviours. Although controls for personal wellbeing, impulsivity, and alcohol consumption were included in the model, along with broader online behaviours, a wider and more in-depth range of covariates were not included, which might have affected our results. Suicide attempts are relatively uncommon, and the number of participants reporting attempted suicide was relatively small for detailed analysis, although larger than in previous studies.^8^, ^37^

Few studies have focused on suicide attempts and problem gambling among young people, and this is the first of our knowledge to be based in Great Britain. Because we used a non-probability sample, replication is required to verify the results. However, the results are consistent with those for adults of all ages recruited using probability methods, while also showing substantial effect sizes for young people.^8^, ^37^

In conclusion, problem gambling could be a substantial risk factor for suicide attempts among both young men and young women. Though this association might be explained in part by a common aetiology such as impulsive or risk-taking personality type, the association persisted even after adjusting for these factors. It is plausible that the severity and multiplicity of harms to relationships, health, and financial security experienced by some who gamble might prompt them to attempt to take their own lives. Gambling researchers have emphasised the need to focus on understanding and articulating the broader range of harms associated with gambling and the scale and nature of these harms, recognising that these harms extend far wider than those currently identified within diagnostic behavioural screens.^42^, ^43^ These data emphasise the harms associated with gambling behaviours for some people, providing evidence for the associations between behaviours and harmful outcomes—specifically between problematic gambling behaviours and suicidality. Further research is needed to understand this association in the context of the rapidly changing gambling environment. In the meantime, young men and young women with problem-gambling behaviours should be considered at increased risk of suicide attempts.

## Data sharing

Data will be deposited with the UK Data Archive in 2021 upon completion of the project. Until then, reasonable requests for data access should be submitted to the lead author (HW).

## References

[1] WHO (2019). Suicide in the world: global health estimates. https://www.who.int/publications/i/item/suicide-in-the-world.

[2] Bould H, Mars B, Moran P, Biddle L, Gunnell D (2019). Rising suicide rates among adolescents in England and Wales. Lancet.

[3] Morgan C, Webb RT, Carr MJ (2017). Incidence, clinical management, and mortality risk following self harm among children and adolescents: cohort study in primary care. BMJ.

[4] Griffin E, McMahon E, McNicholas F, Corcoran P, Perry IJ, Arensman E (2018). Increasing rates of self-harm among children, adolescents and young adults: a 10-year national registry study 2007–2016. Soc Psychiatry Psychiatr Epidemiol.

[5] Gardner W, Pajer K, Cloutier P (2019). Changing rates of self-harm and mental disorders by sex in youths presenting to Ontario emergency Departments: repeated cross-sectional study. Can J Psychiatry.

[6] Rodway C, Tham SG, Ibrahim S (2016). Suicide in children and young people in England: a consecutive case series. Lancet Psychiatry.

[7] Gunnell D, Appleby L, Arensman E (2020). Suicide risk and prevention during the COVID-19 pandemic. Lancet Psychiatry.

[8] Wardle H, John A, Dymond S, McManus S (2020). Problem gambling and suicidality in England: secondary analysis of a representative cross-sectional survey. Public Health.

[9] Fröberg F, Hallqvist J, Tengström A (2013). Psychosocial health and gambling problems among men and women aged 16–24 years in the Swedish National Public Health Survey. Eur J Public Health.

[10] George S, Ts J, Nair S (2016). A cross-sectional study of problem gambling and its correlates among college students in south India. BJPsych Open.

[11] Grant JE, Derbyshire K, Leppink E, Chamberlain SR (2014). Suicidality in non-treatment seeking young adults with subsyndromal gambling disorder. Psychiatr Q.

[12] Cook S, Turner NE, Ballon B (2015). Problem gambling among Ontario students: associations with substance abuse, mental health problems, suicide attempts, and delinquent behaviours. J Gambl Stud.

[13] Stuhldreher WL, Stuhldreher TJ, Forrest KY (2007). Gambling as an emerging health problem on campus. J Am Coll Health.

[14] Jang SM, Hong S (2018). Do addictive behaviours matter for college students' depressive and suicidal ideation?. Int J Ment Health Addict.

[15] Medeiros GC, Sampaio DG, Leppink EW, Chamberlain SR, Grant JE (2016). Anxiety, gambling activity, and neurocognition: a dimensional approach to a non-treatment-seeking sample. J Behav Addict.

[16] McManus S, Gunnell D, Cooper C (2019). Prevalence of non-suicidal self-harm and service contact in England, 2000–14: repeated cross-sectional surveys of the general population. Lancet Psychiatry.

[17] Ng Fat L, Shelton N, Cable N (2018). Investigating the growing trend of non-drinking among young people; analysis of repeated cross-sectional surveys in England 2005–2015. BMC Public Health.

[18] NHS Digital (2019). Health Survey for England 2018. https://digital.nhs.uk/data-and-information/publications/statistical/health-survey-for-england/2018.

[19] NHS Digital (2019). Smoking, Drinking and Drug Use among Young People in England 2018. https://digital.nhs.uk/data-and-information/publications/statistical/smoking-drinking-and-drug-use-among-young-people-in-england/2018.

[20] Connolly A, Davies B, Fuller E, Heinze N, Wardle H (2018). Gambling behaviour in Great Britain in 2016.

[21] Gambling Commission (2019). Gambling industry statistics: April 2015 to March 2019. https://www.gamblingcommission.gov.uk/pdf/survey-data/gambling-industry-statistics.pdf.

[22] NHS Digital (2019). Health Survey for England 2018: supplementary analysis on gambling. https://digital.nhs.uk/data-and-information/publications/statistical/health-survey-for-england/2018/health-survey-for-england-2018-supplementary-analysis-on-gambling.

[23] Abbott M, Binde P, Clark L (2018). Conceptual framework of harmful gambling: an international collaboration.

[24] Twyman J (2008). Getting it right: YouGov and online survey research in Britain. J Elections Public Opin Parties.

[25] Kennedy C, Mercer A, Keeter S, Hatley N, McGeeney K, Gimenez A (2016). Evaluating online nonprobability surveys. https://www.pewresearch.org/methods/2016/05/02/evaluating-online-nonprobability-surveys.

[26] Wardle H (2020). The Emerging Adults Gambling Survey: study protocol. Wellcome Open Res.

[27] McManus S, Bebbington PE, Jenkins R (2020). Data resource profile: Adult Psychiatric Morbidity Survey (APMS). Int J Epidemiol.

[28] Moghaddam JF, Yoon G, Dickerson DL, Kim SW, Westermeyer J (2015). Suicidal ideation and suicide attempts in five groups with different severities of gambling: findings from the National Epidemiologic Survey on Alcohol and Related Conditions. Am J Addict.

[29] Ferris J, Wynne H (2001). The Canadian problem gambling index: final report.

[30] Eysenck SB, Eysenck HJ (1977). The place of impulsiveness in a dimensional system of personality description. Br J Soc Clin Psychol.

[31] Wills TA, Windle M, Cleary SD (1998). Temperament and novelty seeking in adolescent substance use: convergence of dimensions of temperament with constructs from Cloninger's theory. J Pers Soc Psychol.

[32] Auger N, Lo E, Cantinotti M, O'Loughlin J (2010). Impulsivity and socio-economic status interact to increase the risk of gambling onset among youth. Addiction.

[33] Office for National Statistics (2016). Personal well-being in the UK.

[34] Canagasaby A, Vinson DC (2005). Screening for hazardous or harmful drinking using one or two quantity-frequency questions. Alcohol Alcohol.

[35] Tyrer P, Nur U, Crawford M (2005). The Social Functioning Questionnaire: a rapid and robust measure of perceived functioning. Int J Soc Psychiatry.

[36] McManus S, Bebbington P, Jenkins R, Brugha T (2016). Mental health and wellbeing in England: Adult Psychiatric Morbidity Survey 2014.

[37] Cowlishaw S, Kessler D (2016). Problem gambling in the UK: implications for health, psychosocial adjustment and health care utilization. Eur Addict Res.

[38] Mansfield ER, Helms BP (1982). Detecting multicollinearity. Am Stat.

[39] Rao JNK, Scott AJ (1984). On chi-squared test for multi-contingency tables with cell proportions estimated from survey data. Ann Stat.

[40] Mars B, Heron J, Crane C (2014). Differences in risk factors for self-harm with and without suicidal intent: findings from the ALSPAC cohort. J Affect Disord.

[41] Callegaro M, Villar A, Yeager D, Krosnick JA, Callegaro M, Baker R, Bethlehem J, Goritz A, Krosnick JA, Lavrakas P (2014). A critical review of studies investigating the quality of data obtained with online panels. Online panel research: a data quality perspective.

[42] Blaszczynski A, Swanton TB, Gainsbury SM (2020). Avoiding use of stigmatising descriptors in gambling studies. Int Gambl Stud.

[43] Browne M, Langham E, Rawat V (2016). Assessing gambling-related harm in Victoria: a public health perspective.

