# Supplementary material for: Suicidality and gambling among young adults in Great Britain: results from a cross-sectional online survey
Source: Lancet Public Health. 2021 Jan 5;6(1):e39–49. doi: 10.1016/S2468-2667(20)30232-2 (PMC7788474; doi:10.1016/S2468-2667(20)30232-2)
Supplement: Supplementary appendix [file mmc1.pdf]

THE LANCET
Public Health

Supplementary appendix

This appendix formed part of the original submission and has been peer reviewed. We post it as supplied by the authors.

Supplement to: Wardle H, McManus S. Suicidality and gambling among young adults in Great Britain: results from a cross-sectional online survey. *Lancet Public Health* 2021; **6:** e39–49.

Supplementary Material

**Table S1: Profile of respondents to the Health Survey for England, 2017, the Emerging Adults Gambling Survey and National Statistics data**			
	**Data source**		
	**Health Survey for England 2017 (unweighted)**	**Emerging Adults Gambling Survey (unweighted)**	**National Statistics data**
**Sex***			
Male	45.6%	45.8%	48.7%%
Female	54.4%	54.2%	51.3%%
**Ethnic group (18-24 only)****			
White/White British	83.6%	86.8%	81.5%
Mixed	2.5%	3.6%	3.2%
South Asian	9.2%	7.2%	10.3%
Black	3.9%	1.5%	3.7%
Other	0.8%	0.9%	1.3%
**Region (England only)***			
North East	7.6%	4.3%	5.0%
North West	15.6%	12.1%	13.3%
Yorkshire and the Humber	10.4%	10.8%	10.5%
East Midlands	11.7%	8.6%	9.0%
West Midlands	10.4%	9.7%	11.1%
East of England	13.1%	8.9%	10.1%
London	12.8%	17.1%	15.3%
South East	12.1%	16.4%	15.9%
South West	6.3%	12.1%	9.7%
**Economic activity***^**			
Not in Education, Employment or Training	3.4%	12.4%	11.5%

*National statistics computed from the Office for National Statistics 2018 Mid-Population Estimates. Available at https://www.ons.gov.uk/peoplepopulationandcommunity/populationandmigration/populationestimates/datasets/populationestimatesforukenglandandwalesscotlandandnorthernireland
**National Statistics estimates computed from 2011 Census, available at https://www.ethnicity-facts-figures.service.gov.uk/uk-population-by-ethnicity/demographics/age-groups/latest#age-profile-by-ethnicity
***National Statistics estimates from Office for National Statistics bulletin: Young People not in education, employment or training, 2019. Available at: https://www.ons.gov.uk/employmentandlabourmarket/peoplenotinwork/unemployment/bulletins/youngpeoplenotineducationemploymentortrainingneet/august2019
^ Because of differences in how the data were captured, it is not possible to provide equivalent estimates of the proportion of young people in each survey who are students, although it is likely that the YouGov panel over-represents student populations, as 39% of those over 18 were categorised as a student.

Table S2: Comparison of key estimates among different surveys				
	Data source			
	**Health Surveys for England and Scotland 2016**	**Emerging Adults Gambling Survey 2019**	**Adult Psychiatric Morbidity Survey 2007**	**Adult Psychiatric Morbidity Survey 2014**
**MEN (aged 16-24)**				
Past year gambling	52%*	45.5%	62.3%**	N/A
**Problem Gambling Severity Index (PGSI) score**				
Non-problem gambling (PGSI score = 0)	86.8%	78·7%	N/A	N/A
Low risk gambling (PSGI score = 1-2)	9.9%	11·6%	N/A	N/A
Moderate risk gambling (PGSI score = 3-7)	2.4%	4·8%	N/A	N/A
Problem gambling (PGSI score = 8+)	0.9%	4·9%	N/A	N/A
**Suicidality**				
Past year suicide thoughts	N/A	34.1%	5.4%	6.4%
Past year suicide attempts	N/A	3.8%	1.0%	1.9%
**WOMEN (aged 16-24)**				
Past year gambling	39%	39.4%	52.7%*	N/A
**Problem Gambling Severity Index (PGSI) score**				
Non-problem gambling (PGSI score = 0)	98.0%	86·4%	N/A	N/A
Low risk gambling (PGSI score = 1-2)	1.4%	8·9%	N/A	N/A
Moderate risk gambling (PGSI score = 3-7)	0.5%	2·2%	N/A	N/A
Problem gambling (PGSI score = 8+)	0.2%	2·6%	N/A	N/A
**Suicidality**				
Past year suicide thoughts	N/A	38.4%	8.5%	10.4%
Past year suicide attempts	N/A	4.3%	2.4%	2.7%

*Data not published to one decimal place
**Differences in past year gambling prevalence are largely attributable to the declining popularity of the National Lottery.

Example of YouGov survey layout

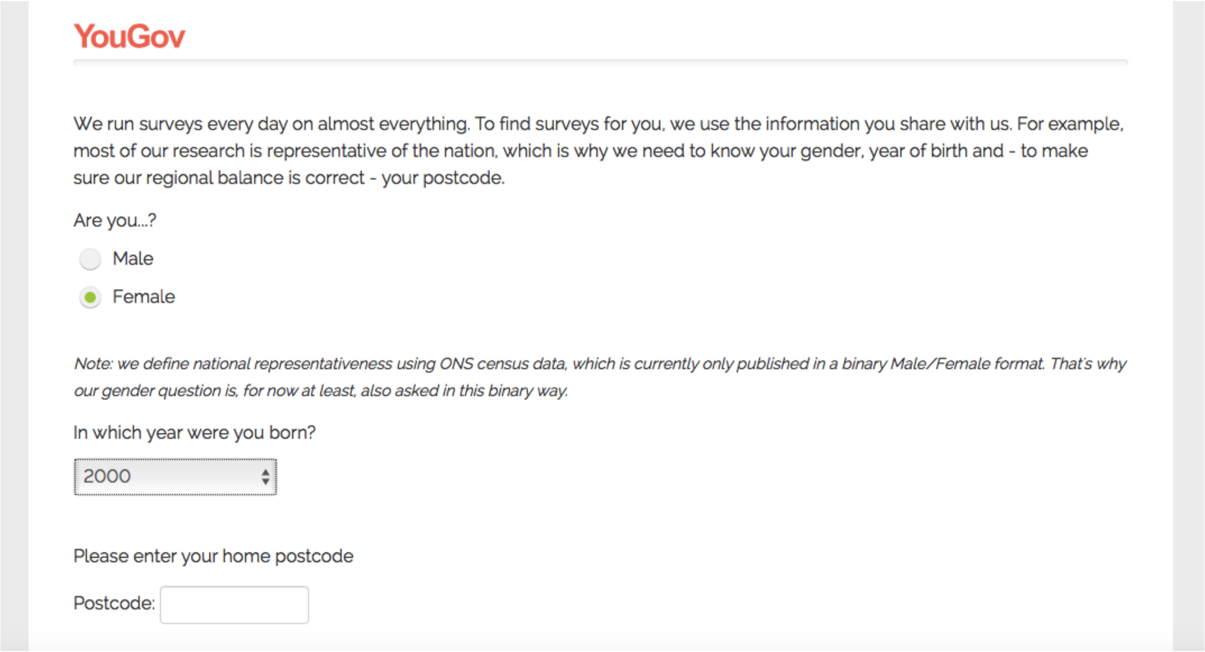

YouGov

We run surveys every day on almost everything. To find surveys for you, we use the information you share with us. For example, most of our research is representative of the nation, which is why we need to know your gender, year of birth and - to make sure our regional balance is correct - your postcode.

Are you...?

Male

Female

Note: we define national representativeness using ONS census data, which is currently only published in a binary Male/Female format. That's why our gender question is, for now at least, also asked in this binary way.

In which year were you born?

2000

Please enter your home postcode

Postcode:

**CHERRIES Item**	**CHERRIES Checklist Category**	**EAGS web-survey compliance**
Design	Describe survey design	The target population was 16 to 24 year old people living in the UK, the sample frame comprised the YouGov panel of over one million people living in Britain. This has up to date information on the profile of each member, allowing subsets of panel members to be invited to participate according to certain characteristics. For this study, participants were eligible if they were aged between 16 and 24, living in Britain and had not taken part in any other YouGov study on gambling in the past year. Only invited panel members were able to take part. (Also see page 4 of the paper).
IRB approval and informed consent process	IRB approval	Institutional Research Board approval was provided by the London School of Hygiene and Tropical Medicine research ethics committee. (Also see page 4 of the paper).
	Informed consent	On YouGov surveys, the panel is informed that participation in any survey they are invited to take part in is voluntary. Participants could only access surveys to which they were both eligible and invited. After acceptance, sufficient information is provided to allow informed consent about undertaking the full study. This included an estimate of the length of time the survey would take to complete. Participants can withdraw from the data collection at any time. Data were initially stored at YouGov, and then provided to the research lead (HW) who stored the data on a secure, password protected server. The dataset passed to the research lead included no personal identifiers. The data will be stored only for as long as needed for the research study. The research investigator (HW) designed the study with the purpose of understanding the context of gambling behaviour among young people in Britain in 2019. (Also see page 4 of the paper).
	Data protection	The data collection was undertaken by one of the largest and most reputable online data collection organisations in the UK. The longstanding panel comprises more than one million UK residents and is maintained by YouGov, who collect and store the personal

CHERRIES Item	CHERRIES Checklist Category	EAGS web-survey compliance
		information needed to assess study eligibility, inform weighting, and study design, and for analytic purposes. Only necessary information for research purposes were shared with the research investigator, who stored the data in secure location to prevent unauthorized access.
Development and pre-testing	Development and testing	The questionnaire was developed by the investigator and reviewed by an expert panel of academics. A field pilot collected data from 62 participants in May 2019 to test the usability and technical functionality of the electronic questionnaire and replicated all procedures used in the main study (including online administration). Pilot responses were reviewed by the investigator and members of the YouGov team and changes agreed. The first 250 responses for the main data collection were further reviewed for consistency, accuracy of routing, and to establish timing thresholds for seriousness checks. (Also see page 4 of the paper).
Recruitment process and description of the sample having access to the questionnaire	Open survey versus closed survey	This was a closed survey, only open to a sample selected, invited and eligible to take part, requiring login, membership verification, and password-protected access.
	Contact mode	Email invites to participate were sent by YouGov to a random selection of their panel members, stratified by region. This email asked them to take part in a survey, without advertising its content, and asked participants to click through to the bespoke study. The first page of the bespoke survey then described the aims and objectives of the survey and obtained informed consent. (Also see page 4 of the paper).
	Advertising the survey	The survey was not open and therefore not advertised.<br><br>The survey introduction for those invited to take part was as follows:

CHERRIES Item	CHERRIES Checklist Category	EAGS web-survey compliance
		**Introduction**<br>You're invited to take part in a research study run by researchers from the London School of Hygiene and Tropical Medicine. Please take time to read the information below about what's involved.<br><br>What's the study about?<br>This study aims to better understand views of betting and gaming among young people and how betting and gaming fits into everyday life and the impact of this. You don't need to have done any betting or gaming to take part, we just want to know what you think.<br><br>What will you be asked to do?<br>You'll be asked about your views on betting and gaming, whether you have taken part in some of these activities and also your use of social media and smartphones. It should take around 10 minutes and you will receive 50 points.<br><br>Can you change your mind and withdraw from the project?<br>Yes. Participation is voluntary so you can opt out at any point by closing your browser.<br><br>**Use of data**<br>Responses from all people who take part will be combined and used to provide statistics showing betting and gaming behaviours of young people. The data provided by YouGov to the research team will be anonymous, and the research team won't know who has taken part. Please see here for privacy notice:<br>https://www.lshtm.ac.uk/aboutus/organisation/data-protection/privacy-notices<br><br>The research team will keep the anonymous data stored securely for up to 10 years. An anonymised version of the data will be provided to the UK Data Archive so that other researchers can use it but this will not contain any personal details that could identify you.<br><br>**What if I have questions?**<br>If you have any questions, please contact Heather Wardle at heather.wardle@lshtm.ac.uk or London School of Hygiene and Tropical Medicine, 15-17 Tavistock Place, London, WC1H 9SH<br><br>**Consent**<br>Please tick the box below to continue<br>1 By ticking this box, you confirm you understand why we are doing the study, that it is voluntary, what will happen afterwards with the information you give, who to contact if you have questions, and that you agree to take part.

CHERRIES Item	CHERRIES Checklist Category	EAGS web-survey compliance
Survey administration	Web/E-mail	The survey was administered on the YouGov online platform. (Also see page 4 of the paper).
	Context	The YouGov panel is marketed as an opinion polling and social, health and market research agency. Recruitment to the panel is open and neutral: it is about sharing opinions and taking part in interesting research. There is also an incentive scheme, whereby participation attracts some renumeration.
	Mandatory/voluntary	It was a voluntary survey.
	Incentives	Participation in YouGov survey is remunerated via YouGov points, which can then be redeemed against rewards offered by YouGov. This survey attracted 50 points, the equivalent cash value of 50p.
	Time/Date	Data were collected between June and August 2019.
	Randomization of items or questionnaires	Generally, because standardised questions and measures were used, and because different participant subgroups were being compared, the item sequence was not randomised. However, certain sets of questions like attitudes to gambling or motivations for gambling were randomised.
	Adaptive questioning	Routing (adaptive questioning) was used to reduce the number and complexity of the questions, minimising participant burden and frustration by ensuring only relevant questions were asked. For example, questions about problem gambling and motivations for gambling were only asked of those who had gambled in the last 12 months.
	Number of items	YouGov has extensive expertise in usability testing and understanding user experience, ensuring the number of questionnaire items per page encouraged full completion.

CHERRIES Item	CHERRIES Checklist Category	EAGS web-survey compliance
	Number of screens (pages)	The full questionnaire was distributed across 64 pages, although due to filtering some participants will have had fewer pages than this.
	Completeness check	All questions were effectively mandatory in that a response had to be submitted in order for the participant to move on. However options were always provided to allow participants to refuse to answer particular items if they choose to, such as a 'not applicable' option and a 'refuse to answer' option. With these included, all responses were complete before the questionnaire was submitted.
	Review step	Respondents were not able to review their responses.
Response rates	Unique site visitor	This issue relates to open access surveys . This survey was closed, and accessible by invitation only.
	View rate (Ratio unique site visitors/unique survey visitors)	This issue relates to open access surveys . This survey was closed, and accessible by invitation only. (Also see page 4 of the paper).
	Participation rate (Ratio unique survey page visitors/agreed to participate)	93% of people who accessed the study introduction page went on to complete the survey. (Also see page 4 of the paper).
	Completion rate (Ratio agreed to participate/finished survey)	The completion rate was 100%; all those who agreed to participate at the informed consent page completed the last question.
Preventing multiple entries from the same individual	Cookies used	This issue is only relevant to open surveys. Duplicate entries were impossible on this study because each survey invite is unique to the panel member.

CHERRIES Item	CHERRIES Checklist Category	EAGS web-survey compliance
	IP check	This issue is crucial for non-panel open access surveys, but not for this study which only recruited unique individual panellists.
	Log file analysis	Multiple entries by one person was not permissible on the system due to restriction to invited participants.
	Registration	As a “closed” (non-open) survey, users needed to login which helps prevent duplicate entries from the same user. The survey never displayed a second time once the user had filled it in.
Analysis	Handling of incomplete questionnaires	Only completed questionnaires were analysed: see section above regarding all questions requiring a response (which could include not applicable or refusal to answer).
	Questionnaires submitted with an atypical timestamp	The time people took to fill in the questionnaire was measured and questionnaires were excluded that were submitted in too short a time (one SD less than the average) or for too long a time (one SD more than the average). In both situations, the exclusions were done due to concern about participant engagement: 39 cases were excluded for this reason. (Also see page 4 of the paper).
	Statistical correction	Weights were developed and applied in analysis to adjust for the non-representative sample. These were produced by the research agency and drew on information about participants and about the population as a whole in relation to age, sex and region. (Also see page 6 of the paper).
